# Further Evidence of an Association between the Presence of *Leishmania RNA Virus 1* and the Mucosal Manifestations in Tegumentary Leishmaniasis Patients

**DOI:** 10.1371/journal.pntd.0004079

**Published:** 2015-09-15

**Authors:** Lilian Motta Cantanhêde, Cipriano Ferreira da Silva Júnior, Marcos Massayuki Ito, Kátia Paula Felipin, Roberto Nicolete, Juan Miguel Villalobos Salcedo, Renato Porrozzi, Elisa Cupolillo, Ricardo de Godoi Mattos Ferreira

**Affiliations:** 1 Fundação Oswaldo Cruz, Unidade Rondonia, Porto Velho, Rondonia, Brazil; 2 Secretaria de Saúde, Governo do Estado de Rondonia, Porto Velho, Rondonia, Brazil; 3 Laboratório de Pesquisa em Leishmaniose, Instituto Oswaldo Cruz, Rio de Janeiro, Brazil; Lancaster University, UNITED KINGDOM

## Abstract

Tegumentary Leishmaniasis (TL) is endemic in Latin America, and Brazil contributes approximately 20 thousand cases per year. The pathogenesis of TL, however, is still not fully understood. Clinical manifestations vary from cutaneous leishmaniasis (CL) to more severe outcomes, such as disseminated leishmaniasis (DL), mucosal leishmaniasis (ML) and diffuse cutaneous leishmaniasis (DCL). Many factors have been associated with the severity of the disease and the development of lesions. Recent studies have reported that the presence of *Leishmania RNA virus 1* infecting *Leishmania* (*Leishmania RNA virus 1*, LRV1) is an important factor associated with the severity of ML in experimental animal models. In the present study, 156 patients who attended Rondonia's Hospital of Tropical Medicine with both leishmaniasis clinical diagnoses (109 CL; 38 ML; 5 CL+ML; 3 DL and 1 DCL) and molecular diagnoses were investigated. The clinical diagnosis were confirmed by PCR by targeting hsp70 and kDNA DNA sequences and the species causing the infection were determined by HSP70 PCR-RFPL. The presence of LVR1 was tested by RT-PCR. Five *Leishmania* species were detected: 121 (77.6%) samples were positive for *Leishmania (Viannia) braziliensis*, 18 (11.5%) were positive for *Leishmania (V*.*) guyanensis*, 3 (1.8%) for *Leishmania (V*.*) lainsoni*, 2 (1.3%) for *Leishmania (Leishmania) amazonensis* and 2 (1.3%) for *Leishmania (V*.*) shawi*. Six (3.9%) samples were positive for *Leishmania sp*. but the species could not be determined, and 4 (2.6%) samples were suggestive of mixed infection by *L*. *(V*.*) braziliensis* and *L*. *(V*.*) guyanensis*. The virus was detected in *L*. *braziliensis* (N = 54), *L*. *guyanensis* (N = 5), *L*. *amazonensis* (N = 2), *L*. *lainsoni* (N = 1) and inconclusive samples (N = 6). Patients presenting with CL+ML, DL and DCL were excluded from further analysis. Association between the presence of the virus and the disease outcome were tested among the remaining 147 patients (CL = 109 and ML = 38). Of them, 71.1% (n = 27) mucosal lesions were positive for LRV1, and 28.9% (n = 11) were negative. In cutaneous lesions, 36.7% (n = 40) were positive and 63.3% (n = 69) were negative for LRV1. The ratio P(ML|LRV1+)/P(ML|LRV1-) was 2.93 (CI95% 1.57…5.46; p<0.001), thus corroborating the hypothesis of the association between LRV1 and the occurrence of mucosal leishmaniasis, as previously described in animal models; it also indicates that LRV1 is not the only factor contributing to the disease outcome.

## Author Summary

Leishmaniasis is an infectious disease caused by protozoa of the genus *Leishmania*. Tegumentary Leishmaniasis (TL) is characterized by a variety of clinical forms, ranging from simple skin lesions to more severe lesions with mucosal destruction. The mechanisms by which some patients develop a severe form of the disease remain unclear. It is already known that the immune statuses of the host and parasite species are key factors influencing the clinical outcome of the disease, but is not known which are the factors that influence the parasite’s tropism to the mucosal regions. Recently, a virus found infecting *Leishmania*, *Leishmania RNA virus 1* (LRV1), has also been reported as one important factor contributing to the disease severity. The present article reports, among 147 investigated patients from the Brazilian Western Amazon, a higher frequency of LRV1 among patients with mucosal lesions compared to patients with cutaneous lesions, statistically demonstrating that the virus is related to the worsening of the disease, increasing the risk of developing mucosal injury by almost 3 times.

## Introduction

Tegumentary Leishmaniasis (TL) is widely distributed worldwide, occurring in 98 countries and affecting approximately 1.5 million people every year. Approximately 90% of the cases are concentrated in 6 countries, including Brazil [1,2].

In Brazil, TL is endemic in all regions, with the highest incidence occurring in the northern region of the country [3]. The Amazon rainforest is located in the northern region and contains a large diversity of parasites, with 7 currently known species of *Leishmania* that causes leishmaniasis, *L*. *(Viannia) braziliensis*, *L*. *(Viannia) guyanensis*, *L*. *(Viannia) lainsoni*, *L*. *(Viannia) naiffi*, *L*. *(Viannia) shawi*, *L*. *(Viannia) lindenbergi and L*. *(Leishmania) amazonensis* [4,5].

In the state of Rondonia, the incidence of TL is high (68.29/100,000), with notifications of approximately 1000 new cases per year [3]. The species *L*. *(Viannia) guyanensis* [6], *L*. *(V*.*) braziliensis*, *L*. *(V*.*) lainsoni* [7], and *L*. *(Leishmania) amazonensis* [8] have been found in Rondonia. However, the frequency of the occurrence of each species is unknown because of the small number of cases studied.

Clinical manifestations of TL can range from simple cutaneous lesions (CL) to more severe presentations that are classified as diffuse (DCL) and mucosal (ML) leishmaniasis. Most authors report that approximately 5 to 10% of both asymptomatic individuals and patients who have been given inappropriate chemotherapy or have recovered from TL can develop ML [9–11]. The number of patients with ML in the northern region is also higher compared with other regions of Brazil, and in Rondonia, approximately 12% of all reported cases are mucosal injuries [3].

The factors involved in the aggravation of TL are still not fully understood. Some authors report that the species of the parasites and vectors, genetic characteristics and immune status of the host [12–15] and, more recently, the presence of *Leishmania RNA virus 1* (LRV1) [16,17] may be related to the diverse clinical outcomes of the disease. This diversity of factors, alone or combined, make understanding the pathogenesis of TL a challenge, particularly in regions such as the Amazon [18].

LRV1 was described primarily in the *L*. *(V*.*) guyanensis* strain that was isolated from a patient with CL. When the strain was inoculated in hamsters, it led to the development of mucosal injury [19], which led to the hypothesis of the importance of viruses in the pathogenesis of the disease. Subsequently, the virus has been described in *L*. *braziliensis* [20], *L*. *major* [21], and recently in *L*. *aethiopica* [22]. Some studies have shown that high loads of LRV1 within parasites was related to the worsening of lesions, which caused tissue destruction due to an exaggerated response of pro-inflammatory cytokines, resulting in a cascading effect from the dsRNA recognition via Toll Like 3 [16,22].

Given the prognostic significance of the *Leishmania* species involved in the infection and the presence of LRV1, our study aimed to identify *Leishmania* species and investigate the presence of LRV1 in patients with cutaneous and mucosal leishmaniasis to provide new evidence on the relationship between both the frequency of the virus and the different species of *Leishmania* with the clinical manifestations of TL.

## Materials and Methods

### Patient recruitment

Patients at the Rondonia Reference Hospital for Tropical Medicine, CEMETRON who were previously diagnosed with leishmaniasis from March 2012 to April 2013 were invited to participate in the study.

CEMETRON Hospital is located in Porto Velho and is a reference center for patient care with various infectious diseases in the state of Rondonia, serving patients from throughout the state and from the border region between the Acre and Amazonas states.

### Ethics statement

The ethical recommendations of the Brazilian National Council of Health were followed with a research protocol registered and approved under the Certificate of Presentation for Ethics Appreciation code CAAE No. 0020.0.046.000–11 by the Ethical and Research Committee of the Center of Research in Tropical Medicine (CEP/CEPEM). Patients who voluntarily agreed to participate in the research were appropriately informed and signed a consent form. Patients were diagnosed according to the recommendations of the Brazilian Ministry of Health [23] routinely applied at the Hospital, which includes clinical and epidemiological criteria. The clinical manifestation of TL was classified as Cutaneous Leishmaniasis (CL), Mucosal Leishmaniasis (ML), concomitant Cutaneous and Mucosal Leishmaniasis (CL+ML), Disseminated Leishmaniasis (DL) and Diffuse Cutaneous Leishmaniasis (DCL). Considering implications that HIV coinfection may have on disease outcome, it is important to notice that following the recommendations of the Brazilian Ministry of Health [23], HIV serological tests are routinely offered to all patients with leishmaniasis. All patients included in the present study were HIV negative.

Samples from the 156 patients diagnosed with TL (109 CL; 38 ML; 5 CL + ML; 3 DL and 1 DCL) were included in the study.

### Collection and processing of samples

Sample collection was performed using a sterile cervical brush placed in direct contact with the internal edge of the lesions. Sampling was performed in duplicate; one sample was used for DNA, and the other was used for RNA extraction. The collected material was immediately stored in an RNAlater solution (Ambion, Austin, TX, USA) for preservation of RNA and was stored at -20°C until the time of analysis. The DNA was extracted using the PureLink DNA Mini Kit (Invitrogen, Carlsbad, CA, USA) according to the protocol established by the manufacturer. The infection was confirmed using a polymerase chain reaction directed toward a fragment of the heat shock protein 70 gene (*HSP70*) [24] and the kinetoplast DNA [25].

**Fig 1:**
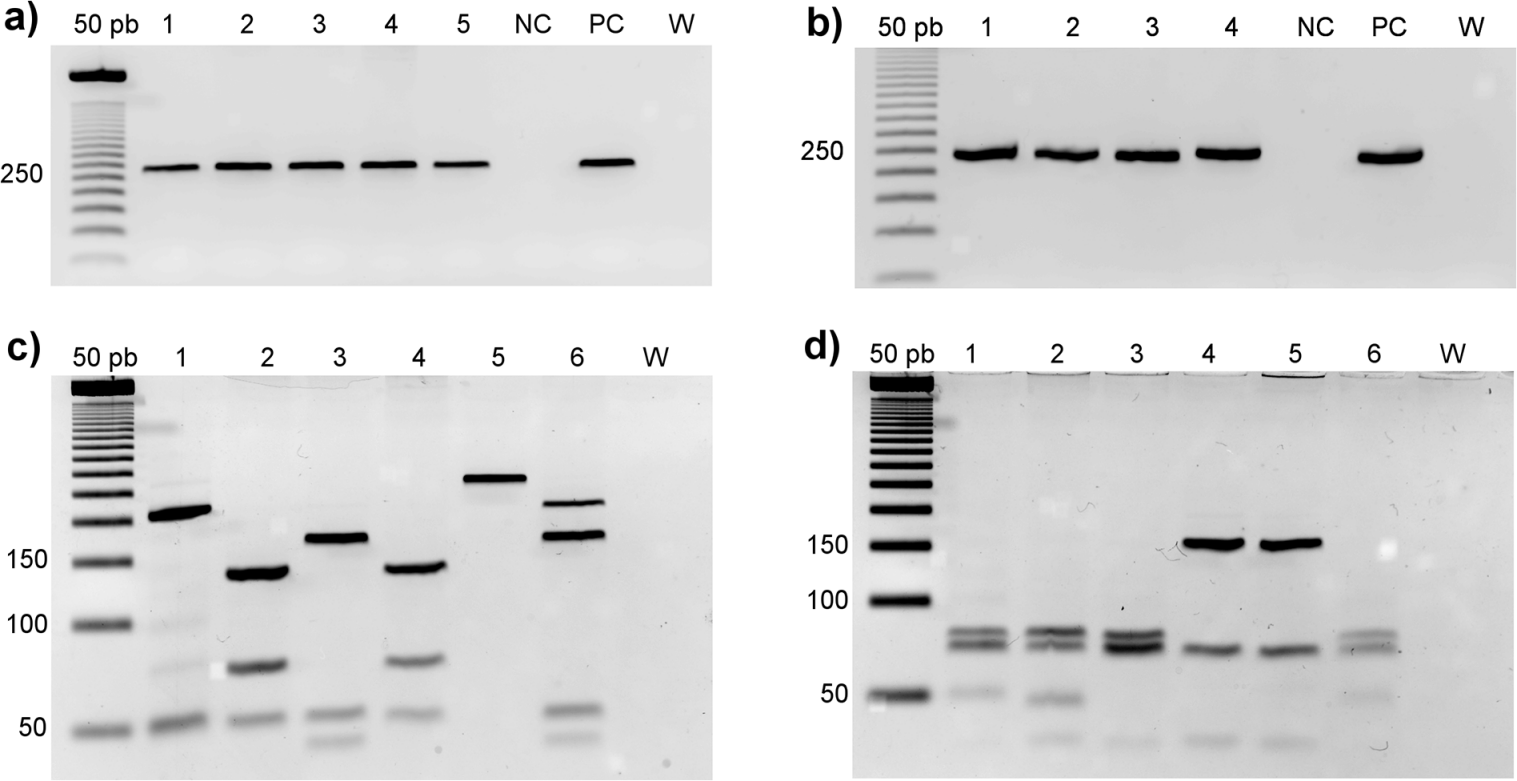
Fragments observed in agarose gels stained with GelRed. Agarose gels [2% for (a) and (b), and 3.5% for (c) and (d)] stained with GelRed. a) PCR product of a fragment of the hsp70 gene of *Leishmania*. 50 bp: molecular weight marker of 50 base pairs; Lines 1–5: clinical samples positive for *Leishmania*; NC: negative control; PC: positive control and W: White. b) RT-PCR amplification of the ORF 1 fragment of LRV1. Lanes 1–4: clinical samples positive for LRV1; NC: negative control; PC: positive control and W: white. PCR-RFLP of hsp70 fragment digested with (c) *HaeIII* and (d) *BstUI*. 1: *L*. *(V*.*) guyanensis*; 2: *L*. *(V*.*) shawi*; 3: *L*. *(V*.*) braziliensis*; 4: *L*. *(V*.*) lainsoni*; 5: L. *(L*.*) amazonensis*; 6: Inconclusive sample and 7: White.

PCR amplifications were performed for hsp70 in a final volume of 50 μL, containing 5 μL of DNA, 0.4 μM of each primer, 200 μM dNTP, 0.75 mM MgCl_2_ and 1 U Taq DNA Polymerase Invitrogen (Life Technologies, Carlsbad, CA, USA). PCR assays were performed using the following cycling: 94°C for 5 minutes followed by 30 cycles at 94°C for 30 seconds, 58°C for 45 seconds and 72°C for 30 seconds and a final extension at 72°C for 5 min. The amplified fragments were visualized on a 2% agarose gel (Fig 1A) stained with GelRed (Biotium, Hayward, CA, USA). For species identification, enzymatic digestion was performed with two restriction enzymes in separate reactions (*HaeIII* and *BstUI*) and was visualized on a 3.5% agarose gel (Fig 1C and 1D respectively) stained with GelRed. Reference strains were employed for comparison of fragment profiles in all PCR-RFLP reactions: *L*. *amazonensis* (IFLA/BR/1967/PH8), *L*. *guyanensis* (MHOM/BR/1975/M4147), and *L*. *braziliensis* (MHOM/BR/1975/M2903). Samples that were not identified by PCR-RFLP were submitted to DNA sequencing for species identification.

**Fig 2:**
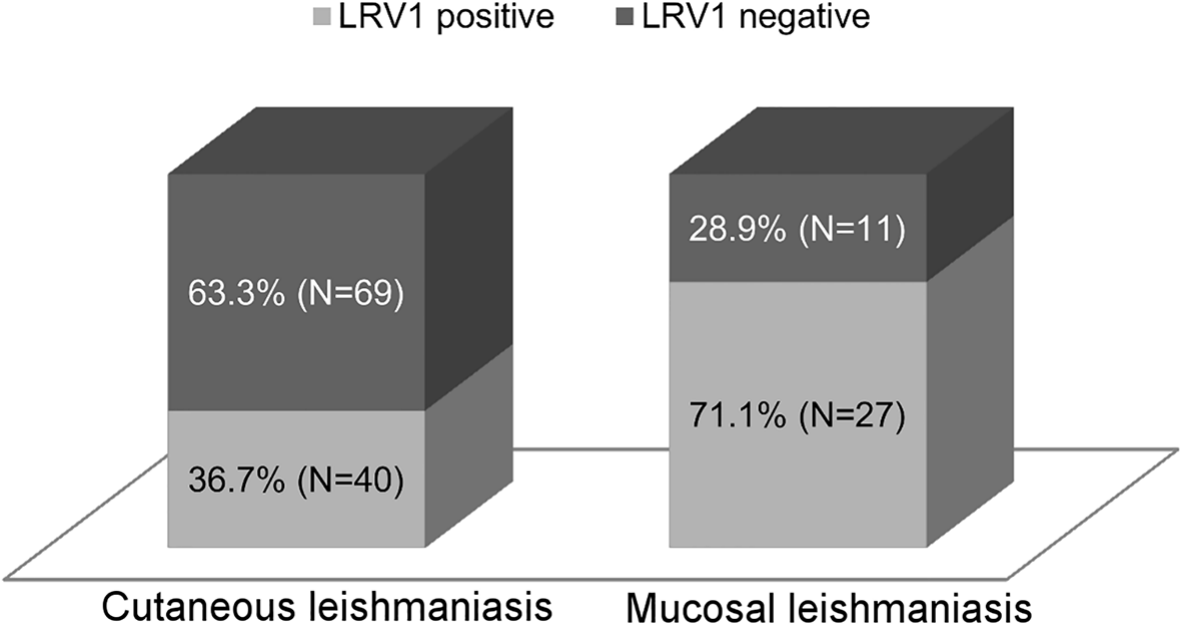
Comparative frequency of LRV1 infection between the two most frequent clinical manifestations of ATL. Frequency of the LRV1 infection is two times greater among ML than CL (p< 0.0001).

The RT-PCR technique was applied for LRV1 detection. Total RNA was extracted using the PureLink RNA Mini Kit (Invitrogen, Carlsbad, CA, USA), and reverse transcription was obtained using SuperScriptIII (Invitrogen, Carlsbad, CA, USA) following the manufacturer's recommendations. The cDNA was submitted to PCR to amplify a 240 bp fragment (LRV1 F– 5’-ATGCCTAAGAGTTTGGATTCG-3’/LRV1 R– 5’-ACAACCAGACGATTGCTGTG-3’) corresponding to the ORF1 region of the viral genome. The forward primer matches exactly the previously described region [26]. The reverse primer was designed using Primer3 [27] and checked for specificity using Primer-BLAST [28]. The amplification reactions were performed in a final volume of 50 μL containing 3 μL of cDNA, 0.2 mM of each primer, 200 μM dNTP, 0.75 mM MgCl_2_ and 1 U Taq DNA Polymerase Invitrogen (Carlsbad, CA, USA) using the following cycling: 94°C for 5 minutes, followed by 30 cycles at 94°C for 20 seconds, 57°C for 15 seconds and 72°C for 30 seconds, and a final extension at 72°C for 5 minutes. The fragments were visualized on a 2% agarose gel (Fig 2B). The cDNA obtained from the strain MHOM/BR/1975/M4147, donated by Coleção de Leishmania do Instituto Oswaldo Cruz (CLIOC), was used as a positive control for all reactions. Some amplicons were sequenced using capillary Sanger sequencing standards protocols to check proper and specific amplification.

### Study design and statistical analyses

A case-control study was conducted to evaluate a possible higher frequency of LRV1 among the ML patients (cases) than among those with CL (controls). It is important to emphasize that CL usually precedes ML in the clinical evolution of the disease; thus, some of the CL patients classified as controls may develop ML in the future. Because LRV1 is a non-infecting virus [29], it was assumed that the Leishmania-virus association was already established before human infection by the parasite. The adopted study design can be classified as conservative because that type of sampling bias decreases the statistical power in finding a significant association.

Data analysis was performed using SPSS version 19. Descriptive analysis of the observed absolute and relative frequencies and their respective confidence intervals of 95% were calculated. The Fisher exact test was applied to evaluate the hypothesis of association using a one-tailed probability. The null hypothesis was that there is no relationship between the presence of LRV1 and the type of injury, and the alternative hypothesis was that LRV1 frequency is increased in patients with mucosal lesion. The relative risk of the presence of LRV1 in patients with ML and 95% confidence intervals were also calculated. The relative risk was defined by the ratio of the probability of the presence of LRV1 in mucosal lesions to the probability of the absence of mucosal lesions in LRV1.

## Results

Samples from the 156 patients diagnosed with TL were included in the study and five different *Leishmania* species were found (Table 1). The most frequent species was *L*. *braziliensis*, with 121 cases (77.6%); 86 patients presented with CL, 28 presented with ML, 5 presented with CL + ML and 2 patients presented with the disseminated form of the disease. *L*. *guyanensis* was detected in 18 (11.5%) patients; 12 presented with CL, and 6 presented with ML. *L*. *lainsoni*, *L*. *amazonensis and L*. *shawi* were the less frequent species and were identified as infecting three (1.8%), two (1.3%), and two (1.3%) patients, respectively. Six (3.9%) samples could not be identified and were classified as *Leishmania sp*. Four samples were suggestive of mixed infection associated with *L*. *guyanensis* and *L*. *braziliensis* after enzymatic digestion and were considered to be inconclusive.

**Table 1:** Observed frequencies of *Leishmania sp*. grouped by disease outcome and presence of LRV1.

Outcome	Species	LRV1		Total
		Positive	Negative	
CL	*Leishmania (V*.*) braziliensis*	33	53	86
	*Leishmania (V*.*) guyanensis*	2	10	12
	*Leishmania (V*.*) lainsoni*	1	2	3
	*Leishmania (V*.*) shawi*	0	2	2
	Inconclusive	3	3	6
ML	*Leishmania (V*.*) braziliensis*	21	7	28
	*Leishmania (V*.*) guyanensis*	3	3	6
	Inconclusive	3	1	4
CL + ML	*Leishmania (V*.*) braziliensis*	2	3	5
DL	*Leishmania (L*.*) amazonensis*	1	0	1
	*Leishmania (V*.*) braziliensis*	1	1	2
DCL	*Leishmania (L*.*) amazonensis*	1	0	1
Total		71	85	156

CL: Cutaneous Leishmaniasis; ML: Mucosal Leishmaniasis; CL + ML: Cutaneous and Mucosal Leishmaniasis; DL: Disseminated Leishmaniasis and DCL: Diffuse Cutaneous Leishmaniasis.

LRV was detected in *L*. *braziliensis* and *L*. *guyanensis* and in *L*. *amazonensis* and *L*. *lainsoni*; thus, this represents the first report, as far as we know, of LRV in *L*. *amazonensis* and *L*. *lainsoni* (Table 1). At least one LRV1 positive sample amplicon of each *Leishmania* species was sequenced to assure the specificity of the amplicon, as well as to provide stronger evidence of the virus presence, considering the differences among sequences. The sequences were deposited at GenBank [30] with the following accession numbers: KT347134, KT347136, KT347137, KT347138, KT347139, KT347140 (from *L*. *braziliensis*), KT34735, KT347141, KT347142 (from *L*. *guyanensis*), KT347143 (from *L*. *lainsoni*) and KT347144 (from *L*. *amazonensis*). Further studies are needed to provide stronger evidence on LRV taxonomy and its relation with *Leishmania* taxonomy. Some samples with unclassifiable *Leishmania* species were also LRV positive.

Patients who showed the least frequent types of injuries, such as CL + ML (n = 5), DL (n = 3) and DCL (n = 1), were excluded from subsequent statistical analyses to avoid confounding factors. Thus, analyses of the association between the presence of the virus and the disease were tested in a total of 147: 109 samples with cutaneous lesions and 38 samples with mucosal lesions, with 36.7% and 71.1% positives for LRV1, respectively.

In total, sixty-seven (45.58%–95%CI 37.7…53.6) samples were positive for LRV1. Forty (59.70%–95%CI 47.7…70.6) of these samples were from cutaneous lesions, and 27 (40.30%–95%CI 29.3…52.2) were from mucosal lesions. Among the 80 negative samples for LRV1, 69 (86.25%–95%CI 76.8…92.3) were from cutaneous lesions, and 11 (13.75%–95%CI 7.6…23.1) were from mucosal lesions (Fig 2).

The relative risk of patients presenting with positive LRV1 mucosal lesions (39.30%) relative to patients presenting with negative LRV1 mucosal lesions (12.30%) was 2.93 (95%CI 1.59…6.36), with Fisher’s exact test of association p-value less than 0.0001.

## Discussion

The results presented herein indicate the diversity of the *Leishmania* species and the complexity of the disease in the studied region. This study reports at least five different *Leishmania* species in Rondonia and a high frequency of LRV1 in cutaneous and mucosal lesions. The *Leishmania* species diversity reported here is similar to that observed in other Amazon regions, but differences in the frequency of each species were observed. In our study, *L*. *braziliensis* was the most frequent species observed associated with human disease, followed by *L*. *guyanensis*. *L*. *lainsoni* and *L*. *amazonensis* were each identified in just one patient. Considering the regions bordering the state of Rondonia, *L*. *braziliensis* was also reported as the most frequent species causing TL in Acre (Brazil), Mato Grosso (Brazil) and Bolivia [31]. However, *L*. *lainsoni* was the second most frequent species; *L*. *naiffi* and *L*. *guyanensis* were less frequently identified as causing TL in humans in Acre [32]. Studies conducted in Bolivia demonstrated that TL is predominantly caused by *L*. *braziliensis* [33,34] and less frequently caused by *L*. *amazonensis* [35], *L*. *lainsoni* and *L*. *guyanensis* [31,36]. There are few studies identifying *Leishmania* species in clinical samples from the state of Mato Grosso, but infections caused by species other than *L*. *braziliensis* are rare; few cases are reported as being caused by *L*. *amazonensis* [37], *L*. *lainsoni* or *L*. *shawi* [24]. A different scenario is observed in the state of Amazonas; *L*. *guyanensis* is the most frequent species causing human CL, followed by *L*. *braziliensis*, *L*. *amazonensis* and *L*. *naiffi* [38].

Mucosal lesions due to *L*. *guyanensis*, a parasite whose distribution is restricted to the Amazon region, have previously been described [39,40]. The present results reinforce the participation of *L*. *guyanensis* as a causative agent of human ML, which was previously commonly associated with *L*. *braziliensis*. The presence of these two etiological agents that are involved with the development of a more severe disease phenotype in the Amazon region can be an important factor in determining the high number of mucosal lesions that are observed in the region [3] in contrast to other Brazilian regions where mucosal lesions are caused exclusively by *L*. *braziliensis*. Another factor that might contribute to the severity of the disease in the Amazon is the high frequency of LRV1 in this region, which is discussed below.

Ten samples showed inconclusive hsp70 RFLP profiles. Of these, four samples were suggestive of mixed infection, presenting fragments corresponding to two different species: *L*. *braziliensis* and *L*. *guyanensis* (Fig 1). The sequences obtained were compatible with either *L*. *braziliensis* or *L*. *guyanensis*. In this case, the PCR product should be cloned before sequencing to evaluate the possibility of mixed infection; this was not performed in this study.

The frequency of LRV is rarely described in clinical samples. The first report of searching for LRV in samples from patients presenting with TL detected two positive cases, among a total of 11 investigated subjects [26]. Another study analyzing samples from TL patients from the Brazilian southeast region analyzed 47 samples and the virus was detected in 12 [41]. In addition to these studies, another study, analyzing biopsy fragments from patients presenting with CL, ML and both forms from different Brazilian regions, focused on patients from the city of Rio de Janeiro (located in the Southeast), where *L*. *braziliensis* is the agent of TL. The virus was detected only in two samples, corresponding to samples from *L*. *guyanensis*-infected patients [42]. Overall, these studies indicate that the severity and chronicity of the disease are not associated with only the presence of LRV. However, not only the presence of LRV but also the LRV burden can have an important role in disease progression (Ives et al 2011).

The role of LRV on the metabolism of *Leishmania* has not been studied in detail, and there is no information concerning why some strains are infected by the virus and others are not [17].

The percentage of ML samples positive for LRV compared with the samples that were negative shows that the presence of the virus contributes significantly to disease aggravation, as previously demonstrated in an animal model [16], but is not a determinant factor in the development of mucosal injury because 28.9% of the samples from mucosal infection showed no LRV infection.

Recently, it was demonstrated that *L*. *guyanensis* infection, regardless of LRV content, induces a Th1 response mediated by MyD88 and TLR-9, two important adaptor proteins from the innate immune response, demonstrating that both proteins are important for the development of healing responses against *L*. *guyanensis* parasites [43]. Patients with mucosal lesions and that are negative for LRV1 may have hyper reactivity, representing an individual predisposition to the disease, whereas those presenting with cutaneous lesions positive for LRV may indicate that each patient will develop a metastatic phenotype dependent on their immunological status and/or time post infection.

Our results indicate that a set of factors must be involved in the aggravation of an initial skin lesion, requiring a combination that has not yet been clarified, and that LVR plays a pivotal role in disease prognosis. Studies of the patients’ immune status correlating the parasite species and presence of LRV1 may clarify the role of the host.

### Conclusions

The parasites isolated in the Amazon region have higher genetic diversity than do isolates from other Brazilian regions [44]. Factors such as the wide geographic distribution, relatively well-conserved forests, a great diversity of natural reservoirs and understudied vectors may contribute to this diversity. The relationship between the genetic variability of *Leishmania spp*. and the high rate of LRV infection in the Amazon needs to be deeply investigated.

Our study showed that LVR infection in the studied region is correlated with the severity of the disease, increasing the relative risk for developing mucosal injury by 2.93 times; however, this influence appears to be limited to the region studied because other studies report that the presence of LRV1 does not correlate with severe chronic TL caused by *L*. *braziliensis* in other endemic areas in Brazil. LRV seems to have a variable contribution to the emergence of mucosal lesions, alone or in conjunction with other factors, such as the genetic characteristics of the host and/or species-specific parasite virulence factors.

Studies with clinical follow-up are needed to confirm the involvement of the virus in the pathogenesis of the disease and the possible role of LRV in the metabolism of the parasite to clarify this unusual symbiosis.

## Supporting Information

S1 Checklist STROBE Checklist. (DOC)
