# Supplementary material for: Further Evidence of an Association between the Presence of Leishmania RNA Virus 1 and the Mucosal Manifestations in Tegumentary Leishmaniasis Patients
Source: PLoS Negl Trop Dis. 2015 Sep 15;9(9):e0004079. doi: 10.1371/journal.pntd.0004079 (PMC4570810; doi:10.1371/journal.pntd.0004079)
Supplement: S1 Checklist — (DOC) [file pntd.0004079.s001.doc]

**Supporting Information**

Checklist S1. In this section information about the adhesion of the current manuscript to the STROBE Statement is provided. The present studied has an important descriptive component over a series of cases. There is not an specific checklist for case-series studies, given that, although the study is not an classical case-control study, the authors evaluated that this is the most suitable check-list to be used. Current lines of the clean version of the article are indicated.

STROBE Statement—Checklist of items that should be included in reports of ***case-control studies***

|  | Item No | Recommendation |
| --- | --- | --- |
| **Title and abstract** | 1 | *(*a) Indicate the study’s design with a commonly used term in the title or the abstract  **The report has an important a case series descriptive component that is clearly described at the abstract and also a hypothesis test as stated in the title, investigating association between two factors. More detailed information about the association is provide (lines 39-41).** |
|  |  | (*b*) Provide in the abstract an informative and balanced summary of what was done and what was found  **The authors evaluated that this recommendation was followed, as can be checked at lines 25-45** |
| Introduction | | |
| Background/rationale | 2 | Explain the scientific background and rationale for the investigation being reported  **The authors evaluated that this recommendation was followed, as can be checked at the Introduction section, from lines 66-107** |
| Objectives | 3 | State specific objectives, including any prespecified hypotheses  **The authors evaluated that this recommendation was followed, as can be checked at the Introduction section, from lines 108-113. Also, in the “Study design and statistical analyses”, the statistical hypothesis under evaluation was precisely defined (lines 201-204).** |
| Methods | | |
| Study design | 4 | Present key elements of study design early in the paper  **Ann specific section “Study design and statistical analyses” (lines 188-197) was created concerning that matter. It is the last Methodology, because the authors evaluated that some methodological definitions should be made before the subsection. If the reviewers or editors recommend and different order, the order of the Methodology subsections can be changed.** |
| Setting | 5 | Describe the setting, locations, and relevant dates, including periods of recruitment, exposure, follow-up, and data collection  **The authors evaluated that this recommendation was followed, as can be checked at lines 116-122.** |
| Participants | 6 | (*a*) Give the eligibility criteria, and the sources and methods of case ascertainment and control selection. Give the rationale for the choice of cases and controls  **For descriptive analysis, the lines 123-134 describes the participants. Regarding cases and controls, additional information is provided in lines 189-197.** |
|  |  | (*b*) For matched studies, give matching criteria and the number of controls per case  **No matching was applied. Given the different time of onset between cases and controls (lines 190-193) and other factors regarding disease progression as well as biological factors of the virus (lines 193-196) the authors found it would be somehow artificial to try to match cases and controls by age.** |
| Variables | 7 | Clearly define all outcomes, exposures, predictors, potential confounders, and effect modifiers. Give diagnostic criteria, if applicable  **The study design is simple, with fill variables assessed aiming to provide relevant and specific information about clinical evidence of association between LRV1 presence and ML as described along Methodology section.** |
| Data sources/ measurement | 8* | For each variable of interest, give sources of data and details of methods of assessment (measurement). Describe comparability of assessment methods if there is more than one group  **Both cases and controls followed similar protocols of assessment.** |
| Bias | 9 | Describe any efforts to address potential sources of bias  **Lines 189 to 197 describe potential bias and how it could affect the results. From the analytical point of view, it is important to emphasize that is well known that ML lesions typically presents much lower parasitemia than CL, what would possibly decrease the sensibility to detect *Leishmania* or LRV1 on ML samples. In that way, analytical error would probably favour the distribution expected over the null hypothesis. The used design was considered by the authors robust to support the conclusions.** |
| Study size | 10 | Explain how the study size was arrived at  **The total number of patients that met the inclusion criteria during the study period (116-118) was included. Since the ML cases were less frequent them CL, cases are less frequent then controls. Considering the main objectives of the study, as well as disease epidemiology, pathology and also the virus biology, the study led to relevant conclusions.** |
| Quantitative variables | 11 | Explain how quantitative variables were handled in the analyses. If applicable, describe which groupings were chosen and why  **Only qualitative variables were studied.** |
| Statistical methods | 12 | (*a*) Describe all statistical methods, including those used to control for confounding  **The statistical methods were describe (lines 198-207).** |
|  |  | (*b*) Describe any methods used to examine subgroups and interactions  **No subgroups or interactions were examined.** |
|  |  | (*c*) Explain how missing data were addressed  **Only fully characterized subjects were included in the manuscript.** |
|  |  | (*d*) If applicable, explain how matching of cases and controls was addressed.  **No matching was applied.** |
|  |  | (*e*) Describe any sensitivity analyses  **No sensitivity analyses was done.** |
| Results | | |
| Participants | 13* | (a) Report numbers of individuals at each stage of study—eg numbers potentially eligible, examined for eligibility, confirmed eligible, included in the study, completing follow-up, and analysed  **Only fully characterized subjects were included in the manuscript.** |
|  |  | (b) Give reasons for non-participation at each stage  **Only fully characterized subjects were included in the manuscript.** |
|  |  | (c) Consider use of a flow diagram  **The authors evaluated that a diagram would not add much information to the manuscript.** |
| Descriptive data | 14* | (a) Give characteristics of study participants (eg demographic, clinical, social) and information on exposures and potential confounders  **Both cases and controls were assessed at the CEMETRON Hospital, a reference center that covers a broad endemic area. Clinical classification is presented at Table I. It is widely accepted that ML usually occurs after CL. Also, infection risks should not affect the analysis, since both cases and controls are infected. Considering the main objectives of the study, as well as disease epidemiology, pathology and also the virus biology, the authors evaluates that all relevant results are presented.** |
|  |  | (b) Indicate number of participants with missing data for each variable of interest  **Only fully characterized subjects were included in the manuscript.** |
| Outcome data | 15* | Report numbers in each exposure category, or summary measures of exposure  **Table 1 provides all the results obtained.** |
| Main results | 16 | (*a*) Give unadjusted estimates and, if applicable, confounder-adjusted estimates and their precision (eg, 95% confidence interval). Make clear which confounders were adjusted for and why they were included  **No adjustments were done. Only exact probabilities were showed.** |
|  |  | (*b*) Report category boundaries when continuous variables were categorized  **No continuous variables were studied.** |
|  |  | (*c*) If relevant, consider translating estimates of relative risk into absolute risk for a meaningful time period  **Do not apply.** |
| Other analyses | 17 | Report other analyses done—eg analyses of subgroups and interactions, and sensitivity analyses  **Do not apply** |
| Discussion | | |
| Key results | 18 | Summarise key results with reference to study objectives  **This study reports at least five different *Leishmania* species in Rondonia and a high frequency of LRV1 in cutaneous and mucosal lesions (lines 243-245). The percentage of ML samples positive for LRV compared with the samples that were negative shows that the presence of the virus contributes significantly to disease aggravation, as previously demonstrated in an animal model (lines 298-300).** |
| Limitations | 19 | Discuss limitations of the study, taking into account sources of potential bias or imprecision. Discuss both direction and magnitude of any potential bias  **This subject is discussed among lines 303 to 316, complementary to what was stated at the Methods section (9^th^ Item of this checklist).** |
| Interpretation | 20 | Give a cautious overall interpretation of results considering objectives, limitations, multiplicity of analyses, results from similar studies, and other relevant evidence  **The authors were very cautious about the study conclusions, emphasizing the further studies are necessary to fully understand the disease clinical spectrum and the role of the virus (mainly lines 318-334).** |
| Generalisability | 21 | Discuss the generalisability (external validity) of the study results  **Given the study objectives, any generalization should be carefully done. The Conclusions section provides a conservative interpretation of the results, and points out that much research needs to be done to clarify these matters.** |
| Other information | | |
| Funding | 22 | Give the source of funding and the role of the funders for the present study and, if applicable, for the original study on which the present article is based  **The funding sources are reported at lines 336-338.** |

*Give information separately for cases and controls.

**Note:** An Explanation and Elaboration article discusses each checklist item and gives methodological background and published examples of transparent reporting. The STROBE checklist is best used in conjunction with this article (freely available on the Web sites of PLoS Medicine at http://www.plosmedicine.org/, Annals of Internal Medicine at http://www.annals.org/, and Epidemiology at http://www.epidem.com/). Information on the STROBE Initiative is available at http://www.strobe-statement.org.
